# 肺癌分子分型与靶向治疗研究进展

**DOI:** 10.3779/j.issn.1009-3419.2012.09.07

**Published:** 2012-09-20

**Authors:** 岚 邵, 正波 宋, 沂平 张, 丹 苏

**Affiliations:** 1 310022 杭州，浙江省肿瘤医院化疗中心 Department of Medical Oncology, Zhejiang Cancer Hospital, Hangzhou 310022, China; 2 310022 杭州，浙江省胸部肿瘤重点实验室 Key Laboratory Diagnosis and Treatment Technology on Thoracic Oncology, Hangzhou 310022, China; 3 310022 杭州，浙江省肿瘤医院肿瘤研究所 Zhejiang Cancer Research Institute, Zhejiang Cancer Hospital, Hangzhou 310022, China

**Keywords:** 肺肿瘤, 分子分型, 靶向治疗, Lung neoplasms, Molecular subtype, Targeted therapy

## Abstract

随着肺癌发生、发展和预后相关分子机制研究的不断深入，肺癌靶向治疗取得了较大的进展，每一种分子分型的发现都会带来相应靶向药物的研究。2004年表皮生长因子受体（epidermal growth factor receptor, *EGFR*）基因在非小细胞肺癌中的发现，为我们带来了*EGFR*突变高度敏感有效的酪氨酸激酶抑制剂（tyrosine kinase inhibitor, TKI）。2007年棘皮类微管相关样蛋白-4-间变型淋巴瘤激酶（*EML4-ALK*）融合基因的出现为肺癌的分子发展带来了新的有效靶点。目前多个肺癌分子靶点及其靶向药物正在研究中。本文旨在回顾并总结肺癌分子分型及其靶向治疗的研究进展。

## Abstract (en)

The discovery of multiple molecular mechanisms underlying the development, progression, and prognosis of lung cancer, has created new opportunities for targeted therapy. Each subtype is associated with molecular tests that define the subtype and drugs that may have potential therapeutic effect on lung cancer. In 2004, mutations in the epidermal growth factor receptor (epidermal growth factor receptor, *EGFR*) gene were discovered in non-small cell lung cancers (NSCLC), especially in adenocarcinomas. And they are strongly associated with sensitivity to EGFR-tyrosine kinase inhibitors (EGFR-TKIs). Moreover, in 2007 the existence of the echinoderm microtubule-associated protein-like 4-anaplastic lymphoma kinase (*EML4-ALK*) fusion gene was discovered in NSCLC, and the same as EGFR-TKIs, ALK inhibitors are being found to be highly effective in lung cancers. At present, multiple molecular subtype of lung cancer and relevant targeted drugs are undering study. Here, we review the remarkable progress in molecular subtype of lung cancer and the related targeted therapy.

肺癌是全球最常见的恶性肿瘤，死亡率居恶性肿瘤之首。我国肺癌发病率呈现逐年上升趋势，年平均增长1.63%^[1]^。近年来，随着基因组、蛋白组学的发展和大量靶向药物的研究和应用，肺癌的治疗已经从传统的标准化“一刀切”治疗模式走上了以基因为导向的个体化治疗之路。肺癌的分子分型在指导临床治疗方案和药物选择以及建立分子分型个体化治疗模式方面扮演着越来越重要的角色。

目前单纯的组织分型已不能满足肺癌个体化治疗的需求，随着分子生物技术的发展，对不同组织亚型的肺癌再进行分子分型，根据肺癌驱动基因突变谱指导分子靶点个体化治疗已经成为了现实。2011年美国临床肿瘤学会（American Society of Clinical Oncology, ASCO）年会上，Mark Kris博士报告了美国国立癌症研究所的肺癌突变联盟开展的一项基于全基因组或多重分子分型的研究结果^[2]^。研究采用了多重检测法对830例肺腺癌肿瘤组织进行10种已知驱动突变（driver mutation）基因的检测，结果显示有60%（252/422）的肿瘤组织有驱动突变，其中KRAS 107例（25%）、EGFR 98例（23%）、ALK重排14例（6%）、BRAF 12例（3%）、PIK3CA 11例（3%）、MET扩增4例（2%）、HER2 3例（1%）、MEK1 2例（0.4%）、NRAS 1例（0.2%）。随后，研究者根据驱动基因突变检测结果对患者进行个性化治疗，如表皮生长因子受体（epidermal growth factor receptor, EGFR）突变患者给予厄洛替尼一线治疗，其它基因突变的则可接受相应的靶向药物治疗或临床试验，对于驱动突变类型尚未确定的患者则予以化疗。Li等^[3]^从202例不吸烟肺腺癌亚洲患者中检测了以下5种突变基因，发现*EGFR*突变152例（75.3%）、*HER2* 12例（6%）、*EML4-ALK* 10例（5%）、*KRAS* 4例（2%）、*ROS1*融合基因2例（1%）。87.1%的患者（176/202）对TKIs治疗敏感，年龄较大的患者*EGFR*突变率较年轻的患者高，年轻的患者上述驱动基因的突变也比较低。

近年来，随着肺癌生物学的飞速发展，针对肺癌重要信号通路和一些关键分子的靶向药物研发也取得了一些新的进展。肺癌生长主要的信号通路是EGFR通路，主要包括RAS-RAF-MAPK和PI3K-AKT，调控细胞的生长、增殖和死亡。主要的靶蛋白包括EGFR、ALK、ROS1、HER2、FGFR1和PDGFRA等，现将肺癌主要的信号通路、靶蛋白及对应靶向药物进展综述如下。

## 1 EGFR

EGFR是一种具有酪氨酸激酶活性的跨膜糖蛋白，为erbB（HER）家族中4个成员之一。非小细胞肺癌（non-small cell lung cancer, NSCLC）中*EGFR*突变率在北美和西欧为10%左右，而在东亚为30%-50%，其中在亚裔、女性、非吸烟、腺癌中EGFR突变率最高，高达70%-80%^[4]^。

### 1.1 EGFR-TKI

肺癌中针对EGFR的小分子酪氨酸激酶抑制剂（tyrosine kinase inhibitor, TKI）吉非替尼、厄洛替尼及埃克替尼都已在中国上市，其有效性主要表现在对外显子19缺失或外显子21 L858R突变的肺腺癌治疗中^[5]^。2004年ISEL研究开启了EGFR-TKI治疗肺癌。随后，全球性多中心BR.21临床研究首次证实厄洛替尼在晚期NSCLC二/三线治疗明显延长总体生存时间（overall survival, OS）（HR=0.73, *P*=0.001），以及腺癌（HR=0.7, *P*=0.008）NSCLC患者的OS。2008年，INTEREST研究^[6]^是第一项在NSCLC二线治疗中开展的EGFR-TKI（易瑞沙）与标准化疗头对头的全球Ⅲ期临床研究，第一次证明了在未经选择的晚期NSCLC二线治疗患者中，EGFR-TKI和标准化疗多西他赛疗效相当，且具有更好的安全性和患者生活质量的改善。2009年，针对亚洲人群的IPASS研究^[7]^改变了肺癌治疗的历史，在优势人群中一线使用EGFR-TKI或化疗得到的总体OS相当，开拓了*EGFR*基因突变晚期NSCLC用一线酪氨酸激酶抑制剂个体化治疗的新时代。随后的OPTIMAL研究是一项旨在比较厄洛替尼单药与吉西他滨加卡铂（gemcitabine+carboplatin, GC）对携带*EGFR*基因敏感突变的中国晚期NSCLC患者的疗效和安全性的Ⅲ期临床研究。2012年ASCO会上最新的数据显示^[8]^厄洛替尼组的中位OS为22.69个月，GC组为28.69个月，厄洛替尼组生存优势不明显（HR=1.04, *P*=0.691, 5）。研究者认为，研究中OS缺乏统计学差异的原因可能在于GC组后续有较高比例的患者交叉接受了EGFR-TKI治疗。后续分析发现同时接受两种方式治疗的患者总生存明显高于仅接受化疗或EGFR-TKI治疗的患者（30.39个月*vs* 11.70个月*vs* 20.67个月，*P* < 0.000, 1）。INFORM、SATURN研究也逐步奠定了EGFR-TKI在NSCLC维持治疗中的地位，吴一龙等^[9]^将SATURN研究中亚洲人群亚组进行了回顾性分析，发现厄洛替尼治疗组无进展生存时间（progression free survival, PFS）明显延长，其*EGFR*突变的OS也有明显延长（*P*=0.023, 3）。2011年Rosell等^[10]^在ASCO年会上报道了一项Ⅲ期临床研究，1, 227例NSCLC患者经过筛选后发现17.5%的患者具有EGFR突变，将其中174例患者随机分到厄洛替尼单药治疗组和一线化疗组（吉西他滨或多西他赛联合铂类），近期结果显示厄洛替尼组PFS明显优于化疗组（9.7个月*vs* 5.2个月，*P* < 0.000, 1），总生存的数据还有待完善。

### 1.2 EGFR-TKI耐药

虽然大多数*EGFR*突变的患者对第一代EGFR-TKIs（吉非替尼或厄洛替尼）都能取得有效性，但还是有一小部分患者原发性耐药，且大多数患者最终会发展为获得性耐药。目前研究发现获得性EGFR-TKIs耐药的主要机制是*T790M*突变和*c-MET*癌基因的扩增。研究^[11]^提示T790M通过引起EGFR空间构像改变、增加EGFR和ATP的亲和力从而削弱TKI和EGFR-TKI区域的结合能力。*c-Met*基因则通过扩增激活ErbB3-PI3K信号途径导致TKI耐药^[12]^。为了解决第一代EGFR-TKIs耐药的问题，目前针对这些机制的第二代EGFR-TKIs已进入了临床试验。主要策略是将EGFR-TKIs与靶标的可逆结合改为共价（不可逆）结合，并扩大了药物结合受体的范围。

Afatinib（BIBW 2992）是高效、口服、不可逆的EGFR和ErbB2-TKI。临床前研究显示afatinib在肺癌模型中高效，包括*EGFR*突变且一代EGFR-TKI可逆结合耐药的。鉴于前期临床研究中的出色数据，该药在2008年2月15日通过美国FDA的快速审批通道。

LUX-Lung 1研究^[13]^入组了585例Ⅲb期/Ⅳ期肺腺癌患者，PS评分0分-2分，接受过1种或2种化疗方案，且接受过厄洛替尼或吉非替尼治疗12周以上，将他们随机按2:1分别进入afatinib（50 mg/d）组或安慰剂组，均接受最佳支持治疗（best supportive care, BSC）。结果显示：中位OS分别为10.8个月和12.0个月（*P*=0.74）。中位PFS分别是3.3个月和1.1个月（*P* < 0.000, 1），主要的毒副反应是腹泻（87%）、皮疹（78%），严重治疗相关毒副反应39例（10%），2例致死。尽管afatinib没有显示出OS的获益（这也与后续治疗的差异有一定的关系），但在PFS上还是有明显的优势。2012年ASCO会上，LUX-Lung 3研究^[14]^是目前最大样本以*EGFR*突变阳性肺癌人群作为研究对象并且以培美曲塞联合顺铂方案作为对照的一线治疗前瞻性研究。入组345例Ⅲb期/Ⅳ期患者，2:1随机分配至afatinib组（A:230）和培美曲塞联合顺铂化疗组（pemetrexed+cis-platinum, PC:115），主要研究终点为PFS。两组基线平衡，中位年龄61岁，女性65%，亚裔72%，从未吸烟68%，EGFR外显子19缺失49%，L858R突变40%，其它突变11%。结果显示，所有患者中afatinib（A）和联合化疗（PC）的中位PFS分别为11.1个月和6.9个月（*P*=0.000, 4）。在308例常见突变患者中（Del 19/L858R），afatinib（A）和联合化疗组的中位PFS分别为13.6个月和6.9个月（*P* < 0.000, 1），客观有效率（objective response rate, ORR）为56%和23%（*P* < 0.000, 1）。afatinib组主要不良反应为腹泻（95%）、皮疹（62%）、甲沟炎（57%）。研究显示afatinib与培美曲塞/联合顺铂化疗相比可以明显延长PFS，不良反应可控。总人群PFS改善4.2个月，常见突变人群（Del19/L858R）PFS改善6.7个月，afatinib可以作为*EGFR*突变肺腺癌一线治疗的合理选择。但就目前的研究中期数据来看，afatinib的ORR和PFS并未超越吉非替尼和厄洛替尼前期所作的临床试验研究结果（IPASS、NEJ002、OPTIMAL、EUTAC研究等），尚期待更多进一步研究的结果。

Dacomitinib（PF299804）是不可逆小分子EGFR-TKIs，研究显示其对EGFR-T790M抑制要高于吉非替尼或厄洛替尼，且对EGFR 20外显子插入突变也有活性。2012年ASCO会上Kris等^[15]^报道了一项Ⅱ期临床试验，研究入组了92例晚期初治NSCLC患者，结果显示dacomitinib一线治疗EGFR外显子19或外显子21突变的肺癌患者部分缓解率（partial response rate, PR）达74%，初步的1年PFS率达77%，初步的中位PFS达17个月。目前研究者正在入组一组*HER2*突变的肺癌患者，期待进一步的研究结果。目前该药物还是临床试验阶段，值得我们进一步的研究。

## 2 EML4-ALK

间变型淋巴瘤激酶（anaplastic lymphoma kinase, ALK）位于2号染色体短臂发生异位重排，与棘皮类微管相关样蛋白-4（EML4）基因融合，形成具有癌基因属性的EML4-ALK。约有4%的NSCLC患者*EML4-ALK*融合基因阳性，患者多为年轻、男性、不吸烟或少量吸烟和腺癌患者^[16, 17]^，通常对化疗和TKI治疗无效，与*EGFR*、*KRAS*突变是互相排斥的，研究发现其在NSCLC中特有。

目前针对该靶点的药物有Crizotinib（PF02341066），2011年8月FDA已经批准该药用于治疗表达*ALK*基因的局部晚期或转移性NSCLC患者。FDA批准crizotinib系基于两项多中心、单组研究结果。这两项研究共纳入了255例局部晚期或转移性的ALK阳性NSCLC患者，一项Ⅱ期临床试验（PROFILE 1005）^[18]^和一项Ⅰ期临床试验（STUDY 1001）^[19]^，主要研究终点均为ORR。在PROFILE 1005（*n*=136）研究中，ORR为50%，包括1例完全缓解（complete response, CR）和67例部分缓解（partial response, PR），疗效持续时间的中位数为41.9周。在STUDY 1001（*n*=119）中，ORR为61%，包括2例CR和69例PR。治疗时间中位数为32周，疗效持续时间的中位数为48.1周。常见的副作用主要有视力障碍、恶心、腹泻、呕吐、水肿与便秘。在两项临床试验中，至少有4%的患者出现3级或4级副作用，包括丙氨酸氨基转移酶升高和中性粒细胞减少。另外，接受crizotinib治疗的患者有可能出现威胁生命的肺炎。Shaw等^[20]^的研究纳入了82例ALK阳性并接受crizotinib治疗的NSCLC患者（治疗组）和36例未接受crizotinib治疗的ALK阳性患者（对照组），2年生存率分别为54%和36%，在文献报道时治疗组的中位OS尚未达到，对照组为20个月。在亚组分析中发现，接受crizotinib二线/三线治疗的30例患者的2年生存率明显高于23例对照者（55% *vs* 12%, *P*=0.004）。在2012年的ASCO会中，报道了关于PROFILE 1005和STUDY 1001研究中疾病进展（progressive disease, PD）患者的后续研究^[21]^，到2011年6月1日止，共有146例患者出现进展。在146例患者中有78例患者（53%）继续服用crizotinib至少2周，进展后crizotinib治疗的中位持续时间为10周。有20例仅有脑部进展的患者（允许同步进行放疗）继续进行治疗（3周-82周）。总结分析：在ALK阳性的NSCLC患者中，服用crizotinib治疗的患者进展时常常为单器官转移。能够继续接受治疗的患者特征为PS评分良好、单器官转移（大多数脑部和肝脏）和先前应用疗效好，当出现进展后应该继续口服crizotinib治疗一段时间。

## 3 KRAS

*KRAS*基因编码位于细胞质内的G蛋白家族，参与生长因子对细胞生长、增殖等调控作用的多个环节。*KRAS*突变主要发现在肺腺癌中，占NSCLC的15%-20%，腺癌的30%-35%，主要是吸烟患者，鳞癌暂时没有突变的报道^[22]^。*KRAS*突变位点在12、13和61密码子，超过90%的突变发生在12密码子。一项研究^[23]^显示*KRAS*突变的发生率有时与*KRAS*基因的增殖有关，同时也被发现与肺癌预后不良相关，*KRAS*突变的肺癌对EGFR-TKI治疗耐药。然而，肺癌中*KRAS*基因突变是否就没有*EGFR*突变这与总生存不相关^[24]^。

目前还没有针对KRAS的有效靶向治疗药物上市，已经有多项临床试验正在进行中，主要针对下游信号通路MAPK和AKT/PI3K。激酶抑制剂（如MEK或MTOR抑制剂）针对RAS下游信号通路可能对*KRAS*突变细胞有效。Selumetinib通过抑制MEK1/2信号转道通路，下调*KRAS*基因的表达。2012年ASCO会上公布了一项随机、双盲Ⅱ期临床研究^[25]^，研究纳入了Ⅲb期-Ⅳ期*KRAS*基因突变的一线化疗后进展的NSCLC患者，分别给予多西他赛（75 mg/m^2^），联合selumetinib（75 mg, bid）或安慰剂。结果显示，相对多西他赛单药组而言，多西他赛联合selumetinib组OS有所延长（9.4个月*vs* 5.2个月），但差异无统计学意义，但ORR（37% *vs* 0, *P* < 0.000, 1）和PFS（5.3个月*vs* 2.1个月，*P*=0.013, 8）均优于多西他赛单药组。同时，联合组3级-4级中性粒细胞减少、乏力、呼吸衰竭及皮疹的发生增加，但两组毒性相关引起的停药事件发生率无明显差异。这是第一个证实多西他赛联合selumetinib在*KRAS*基因变异患者中临床获益的前瞻性研究，填补了在*KRAS*基因突变NSCLC缺乏有效靶向治疗的空白。

## 4 c-MET

*c-MET*是一种编码HGFR蛋白的原癌基因，通常在肺癌中高表达，MET/HGF信号轴与肿瘤细胞的多种生物学功能相关，包括增殖、生存、迁移和侵袭等，同时也是肿瘤血管生成的重要因子。Ma等^[26]^对人肺癌组织标本进行c-MET表达率检测，发现在腺癌、鳞癌、大细胞癌和小细胞癌中的表达率分别为67%、57%、57%和25%。20%的EGFR获得性耐药是由于*MET*基因扩增，在未治疗肺癌患者中只发现2%的扩增。MET在肺癌中有时突变和/或扩增，其扩增率在肺腺癌中约4.1%，是预后不良因素^[27]^。目前MET扩增抑制剂有XL184、ARQ 917-209和Metmab等。ARQ 197-209（tivantinib）是一种选择性、非ATP竞争性c-Met抑制剂，目前还在临床试验中。一项Ⅱ期临床试验^[28]^显示167名患者按1:1随机分成厄洛替尼加ARQ 197（360 mg bid po）（erlotinib+tivantinib，ET组）和厄洛替尼加安慰剂（erlotinib+placebo，EP组），中位PFS分别为3.8个月和2.3个月（*P*=0.24），进一步分析显示EGFR野生型、*KRAS*突变的非鳞癌患者PFS获益最大（*P*=0.006），两组之间毒副反应差别不大，主要为皮疹、腹泻、乏力、恶心和贫血。MetMAb是EGFR/Met的双重抑制剂。一项Ⅱ期研究（OAM4558g）^[29]^显示128例NSCLC患者随机接受MetMAb联合厄洛替尼（MetMAb+erlotinib，ME组）和安慰剂联合厄洛替尼（placebo+erlotinib，PE组）用于二线/三线治疗，其中54%的患者Met表达为阳性，预后不良。结果显示ME组在PFS和OS上都明显好于对照组（PE组）。

## 5 PI3K-AKT-mTOR通路

PI3K-AKT-mTOR通路是EGFR信号通路，主要与抗凋亡相关。细胞外信号分子作用于受体酪氨酸激酶，激活K-ras和磷脂酰肌醇-3激酶（phosphatidylinositol-3 kinase, PI3K），后者通过下游信号作用于mTOR。mTOR是一种丝氨酸/苏氨酸激酶，被认为是一种雷柏霉素的细胞内靶点^[30]^。mTOR通过调节其它激酶，如40S核糖体6激酶（S6k），细胞周期依赖蛋白激酶和真核细胞翻译起始因子的磷酸化，在蛋白质翻译过程中起重要调节作用。虽然与mTOR有关的信号传导途径尚未完全阐明，一个很明显的事实是mTOR参与了蛋白质合成的调节，并与生长因子及其受体、细胞周期进程及膜运输相互作用。

近年来有代表性的mTOR抑制剂包括西罗莫司（Sirolimus）、everolimus（RAD001）和AP23573等，以everolimus为主的基础和临床研究在肺癌上也取得了一定的进展^[31, 32]^。2009年3月30日美国FDA批准everolimus作为二线药物，治疗使用舒尼替尼或索拉非尼无效的晚期肾癌患者，治疗NSCLC处于Ⅱ期临床阶段。Soria等^[33]^进行的一项Ⅱ期临床实验，共入组85例患者，其中一组42例患者既往接受过化疗，另外一组既往行化疗和EGFR-TKI治疗，结果两组中位PFS分别为2.6个月和2.7个月，疾病控制率（disease control rate, DCR）分别为52.4%和42.9%，有效率（response rate, RR）分别为7.1%和2.3%，有趣的是所有取得PR的患者均为既往吸烟的患者。2010年ASCO会上Leighl等^[34]^的一项Ⅱ期临床研究比较了单药厄洛替尼及厄洛替尼联合everolimus在复治晚期NSCLC中的结果。共入组133名患者，结果显示联合组3个月DCR为39.4%，单药组为28.4%，联合组中位PFS为2.9个月，单药组为2.0个月，初步显示出联合治疗较单药治疗的优势。2011年ASCO会上Khuri等^[35]^报道了一项everolimus联合多西他赛在晚期NSCLC患者二/三线治疗的Ⅱ期临床研究，28例患者入组，中位治疗时间为3.5个周期，PR 2例，SD 13例，DCR为53%，中位PFS是2.3个月，中位OS为12个月。*pAkt*基因的高表达与ORR有关（*P*=0.033），但是在PFS（*P*=0.261）和OS（*P*=0.467）方面没有统计学差异。

## 6 VEGF

血管内皮生长因子（vascular endothelial growth factor, VEGF）是一种重要的血管生成因子，其具有增加血管的通透性、促进内皮细胞的增殖、促进血管的生成等重要的生物学功能。莫特沙芬（motesanib）是针对血管内皮生长因子受体（vascular endothelial growth factor receptor, VEGFR）1/2/3、血小板衍生生长因子受体（platelet derivative growth factor receptor, PDGFR）和*KIT*基因的口服血管生成抑制剂。一项Ⅲ期随机双盲、安慰剂对照临床试验（MONET1）^[36]^，按1:1比例将1, 090例进展期非鳞状细胞NSCLC患者随机分为卡铂+紫杉醇+motesanib组（A组）或卡铂+紫杉醇+安慰剂组（B组），直至疾病进展或出现无法耐受的毒性。中位随访10.6个月的结果显示：与B组相比，A组患者的OS（13.0个月*vs* 11.0个月，*P*=0.137）未明显延长，PFS延长了0.2个月（5.6个月*vs* 5.4个月，*P*=0.000, 6）。A组和B组≥3级不良事件的发生率分别为73%和59%，A组较B组更常见的≥3级不良事件为中性粒细胞减少（22% *vs* 15%）、腹泻（9% *vs* 1%）、高血压（7% *vs* 1%）和胆囊炎（3% *vs* 0%）。

贝伐珠单抗是抗VEGF的单克隆抗体，能阻止VEGF与VEGFR结合，从而抑制VEGF的促血管生成活性。2006年美国食品与药物管理局批准贝伐珠单抗联合卡铂和紫杉醇作为晚期非鳞状细胞NSCLC的一线治疗，是目前唯一通过批准的抗血管生成药物。2006年ECOG一项大样本、随机、多中心Ⅲ期随机临床试验（E4599）^[37]^证实贝伐珠单抗联合标准化疗治疗非鳞NSCLC是有效的，878例进展期肺癌患者分别接受紫杉醇和卡铂联合或不联合贝伐珠单抗（15 mg/kg）治疗，结果显示联合组OS明显延长（12.3个月*vs* 10.3个月，*P*=0.003），同时也提高了RR（35% *vs* 15%, *P* < 0.001）、PFS（6.2个月*vs* 4.5个月，*P* < 0.001）。2012年ASCO会上报道的SWOG研究^[38]^是两项分别以78例晚期细支气管肺泡癌患者（S0635）和85例晚期非吸烟肺腺癌患者（S0636）为入选人群的Ⅱ期临床研究，患者每天接受厄洛替尼（150 mg）联合贝伐珠单抗（15 mg/kg），21天重复。结果显示ORR分别为18%和47%，中位OS分别为17个月和26个月，主要毒副反应为皮疹、腹泻和高血压。SWOG研究提示了厄洛替尼联合贝伐珠单抗治疗晚期NSCLC是安全有效的。

## 7 胰岛素样生长因子Ⅰ受体（insulin like growth factor Ⅰ receptor, IGF-IR）

IGF-IR及其配体在肺癌生长中起着关键作用。IGF与受体在细胞层面结合后，激活TK而致胰岛素受体基蛋白磷酸化，使细胞内多种信号通路活化，包括RAS/RAF/MAP激酶及PI3K旁路，从而导致癌基因转化生长和癌细胞存活。有研究证明血浆IGF-1水平升高与肺癌危险性相关。临床前研究显示在NSCLC中IGF信号通路参与肿瘤细胞的增殖、凋亡和侵袭。IGF-1R单抗包括Figitumumab（CP-751, 871）、IMC-A12等目前都还处于临床研究阶段。Ⅱ期临床研究^[39]^入组的156例初治患者随机分成3组，即卡铂联合紫杉醇+figitumumab（10 mg/kg）（paclitaxel+carboplatin+figitumumab, PCF10）、卡铂+紫杉醇+figitumumab（20 mg/kg）（PCF20）和卡铂+紫杉醇（PC）。结果显示在PCF20组中鳞癌患者的DCR达89%，PC组进驻后再接受figitumumab治疗仍有3例鳞癌患者有效，提示figitumumab在鳞癌的治疗中作用明显。但在后期的Ⅲ期研究中却因出现严重的脱水、高糖血症、咯血等毒副反应而提前终止。日本2011年的一项研究^[40]^是figitumumab（20 mg/kg）联合卡铂和紫杉醇在治疗初治NSCLC患者，18例患者获得PR，进一步数据还在分析中。

## 8 融合基因

### 8.1 ROS1

ROS1是58个酪氨酸激酶受体之一。它最早是1987年在恶性胶质瘤中发现，2011年在NSCLC中发现了它。尽管ROS1与ALK的同源氨基酸序列只有49%，但在体外实验中已经证实某些ALK抑制剂能够抑制ROS1的活性。美国学者Bergethon等^[41]^从1, 073例NSCLC患者中检测发现18例（1.7%）的肺癌患者存在*ROS1*基因融合变异，多见于年轻、不吸烟、分期高的患者，均为腺癌。且表达ROS1融合蛋白的HCC78肺癌细胞系或转染表达ROS1的293细胞均对crizotinib非常敏感。2012年ASCO会上crizotinib治疗ROS1阳性的NSCLC研究成为热门，研究共入组15例患者，给予crizotinib（250 mg, bid）治疗，12名患者目前仍在治疗中，对其中14名患者疗效评价，ORR为57.1%，DCR为79%，治疗的中位时间为25.7周。除了轻微的视力障碍外（87%），最常见的不良反应是一过性的谷草转氨酶升高（27%）、腹泻（27%）、低磷血症（27%）、外周性水肿（27%）、谷丙转氨酶升高（20%）、味觉异常（20%）、恶心（20%）、呕吐（20%）、碱性磷酸酶升高（13%）、中性粒细胞减少（13%）和窦性心动过缓（13%）^[42]^。相信crizotinib会成为继吉非替尼、厄洛替尼小分子靶向药物后的又一新起之秀。

### 8.2 RET

2012年韩国Ju等^[43]^在1例*EGFR*、*KRAS*、*ALK*基因野生型的33岁男性非吸烟肺腺癌患者的肝脏转移灶中，第一次发现了*KIF5B-RET*融合变异基因，并进一步从20例*EGFR*、*KRAS*基因野生型肺癌患者中发现了2例含有KIF5B-RET融合变异。美国Lipson等^[44]^从24例肺癌患者中发现1例有KIF5B-RET融合变异。他们通过对常规石蜡包埋组织（FFPE样本）采用靶向捕获再深度测序技术进行分子分型，发现在21个肿瘤相关基因中存在50种变异，高达83%（20例/24例）的肺癌中存在1个-7个驱动分子变异，包括EGFR、KRAS、BRAF、CDK4、JAK2等有潜在靶向研究价值的分子变异。研究者进一步在121例欧洲肺癌样本中发现1例（0.8%），在405例日本及韩国肺癌样本中发现9例（2%）患者存在*KIF5B-RET*基因融合。在所有检测分析的667例肺癌样本中*KIF5B-RET*基因融合频率约为1.8%（12例/667例），而在没有EGFR、KRAS、HER2、ALK、ROS1变异的肺癌中，*RET*基因的融合频率高达6.3%（10例/159例）。体外实验发现多靶点药物如索拉非尼、舒尼替尼及凡德他尼均能抑制该细胞的增殖。日本Kohno等^[45]^从30例日本肺腺癌患者中发现了1例*KIF5B-RET*融合基因，继而在289例肺腺癌中另外发现了5例*KIF5B-RET*融合基因。但是，当研究者检测80例美国肺癌患者及34例挪威肺腺癌患者时，仅从美国曾经吸烟肺腺癌患者中发现了1例。而在日本患者中检出的6例均为非吸烟腺癌，癌组织高表达RET。进一步发现采用转染表达KIF5B-RET融合蛋白的NIH-3T3细胞模型可被双靶点药物凡德他尼抑制生长及体外克隆形成，提示该药具有明显抑制KIF5B-RET融合蛋白活化的作用。日本Takeuchi等^[46]^采用免疫组化或荧光原位杂交技术分别分析了*ALK*、*ROS1*、*RET*这3种融合基因的频率分布。发现在1, 529例肺癌样本中*ALK*、*ROS1*、*KIF5B*融合基因在所有肺癌和肺腺癌中的出现频率分别为3.0%（腺癌中3.9%）、0.9%（腺癌中1.2%）、0.9%（腺癌中1.2%）。同时对1, 116例肺腺癌（71例为融合基因阳性者）进行多因素分析发现，年龄≥50岁、男性、高病理分期和阴性融合基因状态可作为患者预后不良的独立预测因子。

## 9 总结

肿瘤的发生与发展是一个多基因参与的、多步骤的、复杂的生物学过程，通过生物信息学的研究方法利用肿瘤基因进行分子分型来指导临床靶向药物治疗，为癌症治疗开辟了新的道路。上述的EGFR-TKI等靶向药物治疗已经体现了良好的临床效应，逐渐成为NSCLC临床标准治疗的一部分，但还是面临着靶向药物最佳治疗时机，容易耐药，与化疗之间的权衡利弊等尚待解决的问题。目前针对EGFR-TKI耐药的不可逆ErbB家族抑制剂，如afatinib，已经在大型临床试验中获得了良好的结果，有望成为新一代的肺癌靶向治疗药物。如今，针对肺癌分子分型的新的靶向药物的不断上市，如crizotinib等也为肺癌分子靶向治疗不断注入新的活力。RET融合型、ROS1融合型新的肺癌分子亚型的出现，更是为新的分子分型诊治模式的建立带来了新的生机。
